# Subcellular Energetics and Carbon Storage in *Chlamydomonas*

**DOI:** 10.3390/cells8101154

**Published:** 2019-09-26

**Authors:** Adrien Burlacot, Gilles Peltier, Yonghua Li-Beisson

**Affiliations:** Aix Marseille Univ, CEA, CNRS, Institut de Biosciences et Biotechnologies Aix-Marseille, CEA Cadarache CEDEX, 13108 Saint Paul-Lez-Durance, France; adrien.burlacot@cea.fr (A.B.); gilles.peltier@cea.fr (G.P.)

**Keywords:** organelle, chloroplast, mitochondria, peroxisome, starch, oil, reductant, phosphorylating power, photosynthesis, metabolic shuttles

## Abstract

Microalgae have emerged as a promising platform for production of carbon- and energy- rich molecules, notably starch and oil. Establishing an economically viable algal biotechnology sector requires a holistic understanding of algal photosynthesis, physiology, cell cycle and metabolism. Starch/oil productivity is a combined effect of their cellular content and cell division activities. Cell growth, starch and fatty acid synthesis all require carbon building blocks and a source of energy in the form of ATP and NADPH, but with a different requirement in ATP/NADPH ratio. Thus, several cellular mechanisms have been developed by microalgae to balance ATP and NADPH supply which are essentially produced by photosynthesis. Major energy management mechanisms include ATP production by the chloroplast-based cyclic electron flow and NADPH removal by water-water cycles. Furthermore, energetic coupling between chloroplast and other cellular compartments, mitochondria and peroxisome, is increasingly recognized as an important process involved in the chloroplast redox poise. Emerging literature suggests that alterations of energy management pathways affect not only cell fitness and survival, but also influence biomass content and composition. These emerging discoveries are important steps towards diverting algal photosynthetic energy to useful products for biotechnological applications.

## 1. Introduction

**Figure 1:**
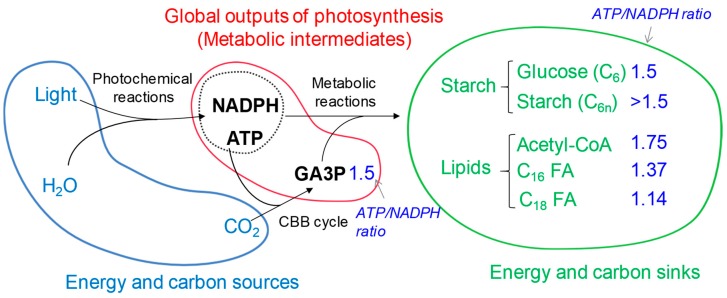
Source and sink relationships in a photoautotrophically grown algal cell. A simple flow is drawn here, but it should bear in mind that catabolisms of «sink» compounds could influence the flux or repartition of «metabolic intermediates». Note: the blue numbers, taken from [2], denote the ratio of ATP/NADPH needed for the production of respective molecules. *Abbreviations:* ATP, adenosine triphosphate; CBB, Calvin-Benson Bassham; CoA, coenzyme A; FA, fatty acid; GA3P, glyceraldehyde-3-phosphate; NAD(P)H, nicotinamide adenine dinucleotide (phosphate).

Microalgae are one of the most diverse eukaryotic organisms occupying wide ecological niches. By converting atmospheric CO_2_ to organic sugars, they provide food to many heterotrophs including humans, and also participate actively in the global carbon cycle [1]. Microalgae perform oxygenic photosynthesis, which occurs in two integrated processes: i) the photochemical light reaction and ii) the Calvin Benson Bassham (CBB) cycle. The photochemical light reactions (linear electron flow–LEF) use solar energy to extract electrons from water and produce reducing equivalent (NADPH). Photosynthesis also generates a proton gradient that is used to produce phosphorylating power (ATP) via the chloroplastic ATP synthase. The chemical energies produced (i.e., NADPH and ATP) are subsequently used to drive CO_2_ assimilation via the CBB cycle. Hence, global outputs of photosynthesis are triose phosphate (i.e., glyceraldehyde 3-phosphate (GA3P)), reducing equivalents (NADPH) and phosphorylating power (ATP), which constitutes the major metabolic intermediates in a cell. These three compounds are essential to support all activities in a cell, i.e., cell division, growth, and reserve accumulation (Figure 1). Microalgae are therefore renewable cell factories that convert solar energy, H_2_O and CO_2_ into organic products. The efficient production, management and partition of the three major photosynthetic products (C, ATP and NADPH) are essential not only to maintain a healthy algal culture, but also key to a sustainable production of algal biomass to meet global demand for food, fuel and other applications.

**Figure 2:**
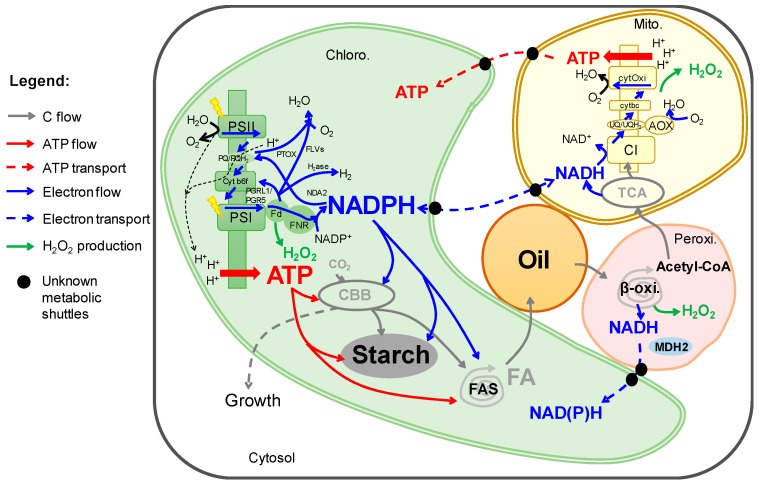
A pathway map showing energy production, management and carbon storage in a *Chlamydomonas reinhardtii* cell. *Abbreviations:* AOX, alternative oxidase; ATP, adenosine triphosphate; β-oxi., β-oxidation; CBB, Calvin Benson Bassham; C1, mitochondria respiratory complex 1; FA, fatty acid; FAS, fatty acid synthase; Fd, ferredoxin; FLV, flavodiiron protein; FNR, ferredoxin-NADP^+^ reductase; Hase, hydrogenase; MDH2, malate dehydrogenase 2; NAD(P)H, nicotinamide adenine dinucleotide (phosphate); NDA2, NAD(P)H dehydrogenase 2; NiR, nitrite reductase; PQ/PQH_2_, plastoquinone/plastoquinol; PTOX, plastoquinone terminal oxidase; PS, photosystem; TAG, triacylglycerol; TCA, tricarboxylic acid cycle; UQ/UQH_2_, ubiquinone/ubiquinol.

Microalgae are able to grow on fluctuating ecological environments and can transiently store energy dense molecules like starch and oil. They have thus recently emerged as a promising platform for production of a range of materials for biotechnological applications [3,4,5]. However, establishing a profitable economy based on algal biotechnology requires a deeper and holistic understanding of algal photosynthesis, metabolism and carbon storage. A fundamental basis for such an understanding is the knowledge on the co-ordination of the production, management and re-distribution of carbon building blocks and energy (ATP and NADPH) between various electron and carbon sinks. ATP and NAD(P)H are essential energy carriers for numerous biochemical reactions occurring in different subcellular compartments. Cell membranes are not permeable to these molecules, therefore coordinating NAD(P)H and ATP levels between various subcellular compartments requires specific trafficking mechanisms [6,7]. A detailed energy and carbon exchange/storage map at a subcellular level in a *Chlamydomonas* cell is drawn in Figure 2. The cellular signal(s) that connect the demand (sink) to the production site (photosynthetic chain, source) are not yet known. Microalgae having impaired energy sinks (starch or oil) usually show lower photosynthetic activity [8,9]. However, we still do not know whether the metabolic demand governs the photosynthetic production of NADPH and ATP, or if it works the other way around.

It should also bear in mind that many of the energy management mechanisms are essential to survival of algal cells in their natural habitats where unfavorable growth conditions are common. However, under optimal laboratory conditions or in well controlled photobioreactors, these control mechanisms are dispensable, and could represent a significant loss in energy. Despite no significant effect on biomass productivity [10], shutting down one or several of these alternative pathways could still be a way to divert more electrons to reserve formation thus enriching energy density of algal biomass.

*Chlamydomonas reinhardtii* (here after abbreviated as *Chlamydomonas*) has originally been developed as a model organism to study photosynthesis using genetic approaches [11], then it has also been used to study cell cycle [12], starch and lately lipid metabolism [13,14,15]. A large indexed mutant library is now available for *Chlamydomonas* [16], which opens unprecedented perspectives for deeper and wider molecular and genetic investigation of algal photosynthesis and metabolism. We review here latest literature on the various energy management pathways identified in *Chlamydomonas* and how the defects or perturbation in one or several of such pathways can impact starch and oil content. The redox biology and photosynthetic efficiencies of *Chlamydomonas* varies if cells are grown in photoautotrophy (with light as the sole source of energy) or mixotrophy (with part of the energy supplied by a reduced carbon source). Acetate is a known organic carbon source for *Chlamydomonas*, its uptake requires ATP and its catabolism produces NADPH [17,18]. Therefore cellular energetic or metabolic context are very different depending on the supply of acetate. We have therefore deliberately chosen to discuss as much as possible scenarios under photoautotrophic conditions, which are relevant to large scale cultivation [19].

## 2. Energy Production and Management in the Chloroplast

### 2.1. Energy Production and Usage

ATP and NADPH are major cellular energy currencies. They are essential to almost all metabolic pathways in the cell. ATP and NADPH are originally produced by light-driven photochemical reactions occurring in the thylakoid membranes. The light-induced oxidation of water by the photosystem II generates the photosynthetic electron flux. Electrons are then funneled towards the cytochrome *b_6_f* complex and photosystem I (PSI) through soluble pools of plastoquinone (PQ) and plastocyanin (PC) respectively acting as membrane and luminal electron carriers. Light energy is eventually used at the PSI level to transfer the photosynthetic electron flux to stromal ferredoxin (Fd). Reduced Fd supplies electrons to the ferredoxin-NADP^+^ reductase (FNR) for NADPH synthesis [20,21] (Figure 2). This photosynthetic electron flow is the so-called LEF. At the level of the cytochrome *b_6_f* (cyt *b_6_f*) the LEF is accompanied by a transfer of protons from the thylakoid stroma to its luminal side. The proton gradient generated is used to fuel ATP synthesis through the plastidial ATP synthase complex. LEF is highly energetic, although it is still a matter of debate, LEF is generally assumed to produce ATP/NADPH at a ratio of 2.5/2 [22].

ATP and NADPH are subsequently used by the CBB cycle to fuel CO_2_ fixation. Efficient use of NADPH generated by the LEF is critical to cell survival and fitness. Indeed, over-reducing pressure in the chloroplast can lead to the production of harmful reactive oxygen species (ROS) causing oxidative damage [23,24,25]. Efficient carbon fixation therefore relies on a proper balance between energy consumption by metabolism and energy supply by photosynthesis. However, since the CBB requires an ATP/NADPH ratio of 3/2 (Figure 1), the LEF production (with an ATP/NADPH ratio of 2.5/2) is short in ATP. In addition, metabolic reactions are slow to acclimate to varying energy inputs which is not the case of photosynthesis. Hence, excessive light or rapid shifts of illumination may result in an imbalance between reducing power produced by photosynthesis and reducing power required by metabolic reactions. To face the potentially harmful energetic imbalance, the LEF is complemented with different mechanisms known as alternative electron flows allowing to i, evacuate the excess reducing equivalent and/or ii, produce extra ATP [26,27]. Although a number of mechanisms of alternative electron flow have been described, their respective contributions in different physiological situations and how they are regulated remains to be explored.

### 2.2. Relationships between Alternative Electron Pathways and Carbon Storage

Coordinating NADPH production to meet cellular metabolic needs is a major task, and algae have developed several pathways to optimize the stoichiometric ratio of ATP and NADPH required for optimal cell functioning in a fluctuating environment. Various ways of dissipation or usage of the photosynthetic reducing power are collectively called “alternative pathways”, which include ‘cyclic electron flow - CEF‘ and oxygen (O_2_) photoreduction (Mehler reaction, plastid terminal oxidase (PTOX), flavodiiron protein (FLV), photorespiration)(reviewed in detail in [10,26,27]). In summary, the known mechanisms have been historically referred as cyclic and pseudo-cyclic photophosphorylations. Both electron transfer pathways generate a proton gradient and therefore generate ATP, cyclic electron flow (CEF) via a recycling of NADPH around PSI and the O_2_ photoreduction via the consumption of NADPH. A brief description of these pathways, and their relationships with metabolic reactions of carbon storage are described below.

#### 2.2.1. Cyclic Electron Flow (CEF)

CEF occurs during photosynthesis and recycles reducing equivalents from the PSI acceptor side to the cyt *b_6_f* complex [10]. By doing so, it generates an additional electron flow, thus increasing the proton gradient generated by cyt *b_6_f*, and resulting in a higher ATP production. Two molecular mechanisms mediate the CEF in *Chlamydomonas*: i) the membrane-associated PGR5/PRGL1 pathway that uses reduced Fd as the electron donor [28,29]; ii) the soluble NAD(P)H dehydrogenase (NDA2) pathway that uses NAD(P)H as the electron donor [30]. The latter is also involved in the functioning of chlororespiration [30,31,32]. A deep remodeling of the photosynthetic apparatus including a decrease of linear electron flow components and an increase in chlororespiration was early reported in *Chlamydomonas* [33] under nitrogen (N) starvation. It was recently shown that NDA2 activity increases under N starvation, and it was concluded that NDA2 plays a critical role during the acclimation of *Chlamydomonas* to N deprivation [34]. While no major change on PGRL1 was observed in the above work (photoautotrophic condition), it was reported by others that the *pgrl1* mutant deficient in the PGR5/PRGL1 mediated CEF accumulates significantly less oil than its parental line during mixotrophic N starvation [35]. This was interpreted as due to the lack of ATP supply to fatty acid synthesis in the mutant [35]. Clearly both NDA2 and PGRL1/PGR5 CEF pathways are involved in the dynamic remodeling of photosynthesis occurring during N-deprivation, and as a major source of energies for all metabolic reactions, it would be interesting to investigate in the future the relationships between these pathways and the energetics of carbon storage in photoautotrophic conditions.

#### 2.2.2. Chlororespiration and Oxygen Photoreduction

O_2_ is a major product of oxygenic photosynthesis, but it is also a major electron acceptor. While photorespiration is the main O_2_-consumption mechanism in C3 plants [36], microalgal photorespiration shows a low activity due both to a lower oxygenase activity of RuBisCO and the presence of a highly efficient CO_2_ concentrating mechanism [37]. Several other reactions can also use O_2_ as an electron acceptor, generally leading to a final production of H_2_O (potentially after a cascade of enzymatic reactions) and being therefore referred as “water-water cycles” [26]. Known mechanisms of O_2_ photoreduction can be either enzymatic reactions catalyzed i, by PTOX [38] that uses electrons from reduced plastoquinol (PQH_2_) to produce H_2_O and is involved in chlororespiration [31,39]; ii, by FLVs [40,41,42] that produce H_2_O using reducing equivalent downstream PSI, or non-enzymatic, and are then referred as Mehler reactions producing reactive oxygen species at the acceptor side of PSI [43]. FLV-mediated O_2_ photoreduction has been recently evidenced in *Chlamydomonas* [41,42] as previously observed in cyanobacteria [24]. FLV is found extremely important for the acclimation to fluctuating light environments [41], most likely because of their ability to manage excess reduction pressure in these conditions. FLVs appear also important in nutrient deficient conditions [44]. Lately, it is shown that in the starch-less mutant *sta6* deficient in the small subunit of the ADP-glucose pyrophosphorylase (AGPase), a greater proportion of the electrons is directed toward O_2_ reduction [42]. However, we still do not know whether starch amount increases in the absence of FLV, and possible interactions between O_2_ photoreduction mechanisms and lipid metabolism remain to be studied.

#### 2.2.3. Hydrogen Production

Under anaerobic conditions, protons (H^+^) can be used as an alternative photosynthetic electron acceptors upon induction of the [Fe-Fe] hydrogenase, thus resulting in the production of dihydrogen (H_2_) from reduced ferredoxin [45]. In the recent years, interaction between electron sinks and H_2_ photoproduction has been studied in conditions of sulfur (S) deficiency in the presence of acetate [46,47]. Upon S-deprivation, *Chlamydomonas* cells accumulate substantial amounts of starch [48] and lipids [49], which are degraded with the onset of anaerobiosis and H_2_ production. Electrons used for H_2_ photoproduction both come from PSII activity and a non-photochemical reduction of PQ [50]. The starchless *Chlamydomona*s mutant *sta6* showed an impaired H_2_ photoproduction upon PSII inhibition [51] indicating that starch breakdown supplies electrons to the PSII independent H_2_ photoproduction [52]. In these conditions, the H_2_ photoproduction may be used as a means to get rid of reductive power produced by carbon storage breakdown, the electron transfer being mediated by an NDA2-dependent pathway [53]. Above studies were mostly carried out in mixotrophic conditions, i.e., in the absence of acetate. Fouchard et al have however shown that similar rates of H_2_ production could be obtained under photoautotrophic conditions with a concomitant starch breakdown [54]. The role of lipid breakdown on H_2_ photoproduction is not yet investigated but probably very minor [47]. 

## 3. Energetic Coupling between Mitochondria and Chloroplasts

As well as the chloroplast-based reactions, exchanges of energy between chloroplast and mitochondria, or with peroxisome have started to be appreciated. Together with the chloroplast, mitochondrion is another major site where ATP is produced [55]. ATP production by the mitochondrial electron transport chain is powered by NADH and sugar oxidation through the tricarboxylic acid cycle (TCA). A communication between mitochondrial respiration and photosynthesis has long been evidenced by the use of respiration inhibitors or by the analyses of photosynthetic performance of genetic mutants defected in components of mitochondrial respiratory chain [56,57,58,59]. Both ATP and NAD(P)H are now generally accepted to be able to commute between the two subcellular compartments i.e., chloroplast and mitochondria, by means of metabolic shuttles. Several shuttles have been proposed, but none of them has been characterized in detail. Possible candidates include the ADP/ATP translocator, malate/oxaloacetate or malate/aspartate shuttle [60] or triose phosphate translocator.

### 3.1. NAD(P)H Trafficking between Chloroplast and Mitochondria

Excess NAD(P)H produced in the chloroplast is known to be exported to mitochondria where it can feed the respiratory chain either to produce ATP, or to be used to reduce O_2_ to water by alternative oxidase (AOX). ATP, at least part of it, can then be shuttled back to chloroplast to sustain chloroplast metabolism. This was first evidenced in *Chlamydomonas* by Peltier and Thibault [61], and later observed in the *aox* mutants of *Phaeodactylum tricornutum* [62] and *Chlamydomonas* [58] and also in the *Chlamydomonas pgrl1* mutant with an impaired CEF [28]. Cell membranes are not permeable to NAD(P)H, therefore their transport from one subcellular site to another requires a mediator. Malate dehydrogenase (MDH) has long been postulated to play such a role [6,7]. MDH catalyzes the reversible oxidation of NAD(P)H to NAD(P)^+^, oxidizing malate to oxaloacetate (OAA) in the meantime. Malate or OAA can be shuttled across subcellular membranes by metabolite transporters [6,7]. The *Chlamydomonas* genome encodes five MDHs [63], and with only MDH5 requiring NADPH as a co-factor [64], and the other four (MDH1-4) using NADH. MDH5 is considered chloroplastic, whereas MDH4 predicted as mitochondrial, MDH2 peroxisomal, and MDH3 cytosolic and MDH1 (either chloroplastic or peroxisomal) [65,66,67,68]. Besides the peroxisomal MDH2 (detailed in Section 4), none of the other MDH isoforms has been studied in detail. The MDHs involved in trafficking of reducing equivalents between chloroplast and mitochondria remain to be identified. The molecular identity of the proteins involved in malate transport across membranes of subcellular compartments is another unknown even if a malate shuttle has been identified in the *Chlamydomonas* genome [63].

The reductant exchange between mitochondria and chloroplasts could be bidirectional. It has also been postulated that NAD(P)H could also be transported from mitochondria to chloroplast [7]. This function could be important in activating CBB cycle enzymes prior to photosynthetic production of reductant, therefore allowing CBB cycle operates simultaneous to photochemical production of NADPH. But experimental evidence to support such a hypothesis is still lacking.

### 3.2. ATP Import from Mitochondria to Chloroplast

The presence of an active and functional ATP import pathway from mitochondria to chloroplast has initially been evidenced by the study of the mutant *fud50* deficient in chloroplast ATP synthase [69]. The *fud50* mutant cannot grow photoautotrophically, and a suppressor screen identified two independent mutants (*fud50su*) that have a recovered photoautotrophic growth but without a functional ATP synthase. Further inhibition of mitochondrial activities in the *fud50su* lines again abolished the growth capacity of the *fud50su* strains. This is interpreted as the occurrence of ATP transport from mitochondria to chloroplast, that mitochondria-derived ATP is sufficient to allow regain of photoautotrophic growth of the *fud50su* strains.

Another example of the possible occurrence of an ATP import from mitochondria to chloroplast is the characterization of the *bckdh* mutants deficient in the E1α subunit of the branched-chain ketoacid dehydrogenase (BCKDH), a key enzyme in the catabolism of branched chain amino acids (BCAA) in the mitochondria [70]. The *bckdh* mutants made 30% less oil with a reduced rate of mitochondrial respiration (20–30% less). The reduction in total lipid (including oil content) in the *bckdh* mutants is considered due to a shortage in the supply of acetyl-CoA as well as ATP (which are both products of BCAA catabolism in mitochondria) to de novo fatty acid synthesis in the chloroplast [70].

How do ATP produced by mitochondria-based reactions pass through cytosol and finally get into chloroplast remain unknown. A direct transport is possible since mitochondria are often observed located right next to the chloroplast in some transmission electron micrographs [71]. Candidate genes encoding homologous proteins to known plant ATP/ADP transporters are present in the genome of *Chlamydomonas* [63], but none of these proteins has been characterized yet. Therefore, the route of an ATP import from mitochondria to chloroplast remains speculative.

## 4. Energetic Coupling between Peroxisomes and Chloroplasts

Thus far, in the landscape of subcellular energetics, most literature has focused on chloroplast-based process or its interaction with mitochondria [2,27,72]. In addition to chloroplasts and mitochondria, peroxisomes are a third subcellular compartment involved in energetic metabolism. Its implication in subcellular metabolism of *Chlamydomonas* has started to be appreciated [17,68]. NADH is produced through degradation of fatty acids, collectively called fatty acid β-oxidation [67,73,74]. Through characterization of knock-out mutants of the peroxisomal MDH2 in *Chlamydomonas*, it has been evidenced that NADH issued by fatty acid β-oxidation reactions is communicated to the chloroplast. This exchange of reducing equivalent between peroxisome and chloroplast is mediated by MDH2. In its absence, the *Chlamydomonas mdh2* mutants exhibited better sustained LEF rate and resulted in higher production of NADPH under N deprivation. The increased capacity in production of NADPH led to an increased level of fatty acids and starch in the mutants [74]. MDH2 thus connects peroxisomal fatty acid catabolism to starch and lipid synthesis and photosynthetic electron transport in the chloroplast by transmitting the reducing equivalent from the peroxisome to the chloroplast. This is so far the first and only example of a redox communication from peroxisome to chloroplast. Possible additional involvement of H_2_O_2_ in this communication has been proposed [74]. MDH catalyzed reactions are reversible, and a reversed transmission of reducing equivalent from chloroplast to peroxisome might happen in specific conditions. But for the moment, this remains to be witnessed.

## 5. Energy Storage: Biomass, Starch and Fatty Acid Synthesis

CBB cycle connects energetic metabolism to carbon metabolism. The CBB cycle uses the energy from NADPH and ATP to convert CO_2_ and water into organic compounds that can be integrated into other subcellular metabolisms subsequently. The key enzyme of the cycle is the ribulose-1,5-bisphosphate carboxylase/oxygenase (RuBisCO). The carbohydrate products of the CBB cycle are three-carbon phosphate molecules, or “triose phosphate” namely glyceraldehyde-3-phosphate (GA3P). GA3P is a central metabolite participating in many biosynthetic reactions, for example it can be used either by chloroplast-based reactions or exported to other subcellular compartments to drive anabolic reactions elsewhere. Thanks to their capacity to grow heterotrophically, mutants deficient in the large subunit of RuBisCO - the *rbcL* mutant - have been isolated and characterized [75]. Consistent with its role as a major electron sink, the *rbcL* mutants displayed an aberrant LEF but with enhanced CEF activities [18].

### 5.1. Biomass

For unicellular organisms, biomass accumulation is directly linked to cell division and growth activities. Indeed, cell division is known to be one of the most energy-consuming events during the life of a cell [12]. This is supported by the observation that when N is absent in the media, cell division/growth is arrested, and the carbon and energies harvested through an impaired photosynthesis are shunted toward starch and oil, which are accumulated in large amount in the form of starch granules and lipid droplets [76,77].

### 5.2. Starch Synthesis and Degradation: Its Impact on Electron Fluxes

During photoautotrophic growth, the major outputs of CBB cycle is used partly for cell division and growth, and partly as storage for night-time use when carbon and energy levels are low. The major form of carbon storage during a standard growth in *Chlamydomonas* is starch, a glucose polymer stored in the chloroplast of land plants and most green algae [78]. Starch synthesis occurs during the day and its degradation starts as night falls [79,80]. This strategy has been developed in plant and algal cells to optimize growth and avoid night-time carbon starvation. Mutants deficient in starch synthesis, for example the *sta6* mutant, have been observed to exhibit reduced photosynthetic performance and poor growth [8,18]. Similar to the *rbcL* mutant, CEF and other alternative electron pathways are enhanced in the *bafJ5* mutant [18].

Competition between starch and oil accumulation is therefore at least at three levels i.e., C, ATP and NAD(P)H, which might explain the contrasting findings present in the literature [76,81]. In addition, this difference could also be brought about by different genetic makeup, nutritional and environmental factors (light, CO_2_, acetate). While starch and oil are often considered two similar “sinks” for storing carbon and energy [82], it should be noted here that their differential requirement for ATP and NADPH (Figure 1) may be linked to a distinct physiological function.

### 5.3. De Novo Fatty Acid Synthesis and Requirement for ATP and NADPH

In algae, similar to land plants, de novo fatty acid synthesis occurs in the chloroplast where the redox state is highly dependent on photosynthetic activities and where lipid metabolism intersects starch metabolism. It has been observed that the expression pattern for many genes encoding components of de novo fatty acid synthesis is similar to that of genes encoding photosynthetic peptides in developing seeds of *Arabidopsis thaliana* [83], suggesting that photosynthetic activity in developing seeds contribute to carbon acquisition as well as to the heavy demand by fatty acid synthesis for reductants and ATP. Moreover, several enzymes of initial fatty acid synthesis are likely under redox regulation as suggested by the study of deep thioredoxome in *Chlamydomonas* [84]. Among which, there are an acetyl-CoA synthetase, key components of acetyl-CoA carboxylase (ACCase) as well as those of fatty acid synthase (FAS).

The first and committed step of fatty acid synthesis is the formation of malonyl-CoA from acetyl-CoA catalyzed by acetyl-CoA carboxylase (ACCase). This reaction requires one molecule of ATP [85]. One plastidial and one cytosolic isozyme occur in *Chlamydomonas* [5]. The plastidial isoform is made of four distinct subunits [5,85], and it is known to be under tight redox regulation [86], thereby connecting chloroplast energy status to carbon metabolism. The assembly of a fatty acyl chain is then catalyzed by four distinct enzymatic activities collectively called fatty acid synthase (FAS) [85]. In addition to acetyl-CoA, FAS reaction requires a stoichiometric supply of ATP and NADPH (1/2) (Figure 1). This requirement forms sharp contrast to that optimal for starch synthesis (ATP/NADPH demand is 3/2) (Figure 1). No mutant deficient in components of FAS has been isolated so far from *Chlamydomonas*. Nevertheless, *Arabidopsis* mutants deficient in an enoyl-acyl carrier protein reductase (ER), catalyzing the last reaction in the FAS complex, are defected in fatty acid synthesis and showed programmed cell death as a result of ROS over-production associated with the mitochondrial electron transport [87]. Later on, the same group concluded that a block at this enzymatic step resulted in over-accumulation of NADH, which is then shuttled to mitochondria through the malate valve (the action of a plastid located NAD-dependent malate dehydrogenase) therefore triggering ROS formation [88]. These studies demonstrate well that fatty acid synthesis is a major sink for electrons, and that the redox communication between chloroplast and mitochondria goes beyond the immediate context of photosynthesis. However, the existence of such a mechanism in algae remains to be investigated.

In addition to above key steps of de novo fatty acid synthesis, precursor supply i.e., acetyl-CoA has been increasingly shown to play a critical role in determining cellular oil content [5,70,89]. Under photoautotrophic conditions, pyruvate is usually considered the “major” source of plastidial acetyl-CoA, a reaction catalyzed by pyruvate dehydrogenase (PDH). This energetically costly reaction releases a CO_2_ molecule while generating a NADH. *Chlamydomonas* lines with reduced amount of PDH showed a dramatic decline in photosynthesis under photoautotrophic growth [90]. A shortage in the supply of acetyl-CoA for fatty acid synthesis is shown detrimental to algal physiology (absence of a major electron sink), therefore providing another evidence that TAG synthesis under N starvation acts as an important carbon and energy sink. In addition to pyruvate, the *Chlamydomonas* genome encodes three genes encoding acetyl-CoA synthetase (ACS) [63]. ACS catalyzes the formation of an acetyl-CoA from acetate and this reaction is driven by ATP. Recently, ACS has been shown to play a complementary role in supplying acetyl-CoA destined for fatty acid synthesis in *Chlorella desiccata* [91]. Considering the flexibility of *Chlamydomonas* cells to grow under mixotrophic/heterotrophic conditions, and also the production of acetate by fermentative reactions in the cell, ACS should play an important role in *Chlamydomonas*.

Fatty acids are precursors to all acyl-lipid synthesis, including triacylglycerols (TAGs, or oils). Beyond the steps of de novo fatty acid synthesis, ATP or NADPH contributes to lipid synthesis at four other major areas: i) the activation of acyl chains, for example the reactions catalyzed by long chain acyl-CoA synthetase (LACS) [92,93,94]; ii) the movement of acyl-chains from one compartment to another, which often requires ATP-binding cassette (ABC) transporters where ATP is a key co-factor [95,96]; iii, at the level of fatty acid desaturation reactions which require an electron donor [97,98]; and iv, at the level of glycerol 3-phosphate dehydrogenase (GPDH) which plays a key role in the production of glycerol 3-phosphate (G3P) to initiate glycerolipid assembly. Energy homeostasis and lipid metabolism therefore intersect mostly at the level of fatty acid synthesis.

## 6. Conclusions and Perspectives

Due to two major urgent societal issues, i.e., the need for renewable energies and global warming, research on microalgae has flourished in the last 10–15 years. Large international efforts have since been put in place. Oil, starch, and growth are major electron sinks for a cell, and the diversion of their metabolism to one form or another is governed essentially by the cellular redox poise, which is a collective response of cells’ energy production and management pathways to a fluctuating environment. Novel insights into the energetic requirements and constraints in carbon fixation and storage in *Chlamydomonas* have started to emerge. One notable example to elucidate the importance of the connection between photosynthesis and reserve formation is the study on the *std1* mutant. The *std1* mutant is deficient in a gene encoding a DYRK (a dual-specificity tyrosine-phosphorylation-regulated kinase) kinase, and has a better photosynthetic CO_2_ fixation during N starvation, and as a result, higher amount of biomass, oil and starch is made in this mutant [99]. It is hypothesized that the DYRK kinase acts as a negative regulator of the sink capacity of photosynthetic cells that integrates nutrient and energy signals. Detailed molecular mechanisms that connects carbon and energy metabolisms are currently under investigation in the authors’ laboratory. Taken together, current knowledge suggests that improved understanding of the articulation between photosynthetic carbon fixation and their adaptation to environment is a key to solve fundamental issues in algal biotechnology, and increasing knowledge in this area should aid in the design of future strategies in not only improving biomass production but also altering its composition.

Considering the complex nature of algal biology, an integrated understanding of algal photosynthesis, physiology and metabolism is required. A systematic understanding of the interaction between redox and carbon reserve metabolism is still to come. Such a question becomes possible now to be addressed due to the development of biophysical techniques in NADPH measurement and due to the availability of large number of well characterized genetic mutants defected either in photosynthetic activities [16], in mitochondrial respiratory activities [55,57,100], or in starch/oil accumulation capacities [74,80,99,101]. Combined with novel synthetic biology principals and tools [102,103,104], the future of algal biotechnology looks promising.

## References

[1] Chen Y., Xu C., Vaidyanathan S. (2018). Microalgae: A robust “green bio-bridge” between energy and environment. Crit. Rev. Biotechnol..

[2] Johnson X., Alric J. (2013). Central carbon metabolism and electron transport in *Chlamydomonas reinhardtii*: Metabolic constraints for carbon partitioning between oil and starch. Eukaryot. Cell.

[3] Wijffels R.H., Barbosa M.J. (2010). An outlook on microalgal biofuels. Science.

[4] Merchant S.S., Kropat J., Liu B., Shaw J., Warakanont J. (2012). TAG, You’re it! Chlamydomonas as a reference organism for understanding algal triacylglycerol accumulation. Curr. Opin. Biotechnol..

[5] Li-Beisson Y., Thelen J.J., Fedosejevs E., Harwood J.L. (2019). The lipid biochemistry of eukaryotic algae. Prog. Lipid Res..

[6] Mettler I.J., Beevers H. (1980). Oxidation of NADH in glyoxysomes by a malate-aspartate shuttle. Plant Physiol..

[7] Scheibe R. (2004). Malate valves to balance cellular energy supply. Physiol. Plant.

[8] Work V.H., Radakovits R., Jinkerson R.E., Meuser J.E., Elliott L.G., Vinyard D.J., Laurens L.M.L., Dismukes G.C., Posewitz M.C. (2010). Increased lipid accumulation in the *Chlamydomonas reinhardtii* sta7-10 starchless isoamylase mutant and increased carbohydrate synthesis in complemented strains. Eukaryot. Cell.

[9] Li X., Moellering E.R., Liu B., Johnny C., Fedewa M., Sears B.B., Kuo M.-H., Benning C. (2012). A galactoglycerolipid lipase is required for triacylglycerol accumulation and survival following nitrogen deprivation in *Chlamydomonas reinhardtii*. Plant Cell.

[10] Chaux F., Peltier G., Johnson X. (2015). A security network in PSI photoprotection: Regulation of photosynthetic control, NPQ and O_2_ photoreduction by cyclic electron flow. Front. Plant Sci..

[11] Harris E. (2001). Chlamydomonas as a model organism. Annu. Rev. Plant Physiol. Plant Mol. Biol..

[12] Cross F.R., Umen J.G. (2015). The Chlamydomonas cell cycle. Plant J..

[13] Buleon A., Gallant D.J., Bouchet B., Mouille C., Dhulst C., Kossmann J., Ball S. (1997). Starches from A to C—*Chlamydomonas reinhardtii* as a model microbial system to investigate the biosynthesis of the plant amylopectin crystal. Plant Physiol..

[14] Li-Beisson Y., Beisson F., Riekhof W. (2015). Metabolism of acyl-lipids in *Chlamydomonas reinhardtii*. Plant J..

[15] Riekhof W.R., Sears B.B., Benning C. (2005). Annotation of genes involved in glycerolipid biosynthesis in *Chlamydomonas reinhardtii*: Discovery of the betaine lipid synthase BTA1(Cr). Eukaryot. Cell.

[16] Li X., Zhang R., Patena W., Gang S.S., Blum S.R., Ivanova N., Yue R., Robertson J.M., Lefebvre P.A., Fitz-Gibbon S.T. (2016). An Indexed, Mapped mutant library enables reverse genetics studies of biological processes in *Chlamydomonas reinhardtii*. Plant Cell.

[17] Plancke C., Vigeolas H., Hohner R., Roberty S., Emonds-Alt B., Larosa V., Willamme R., Duby F., Onga Dhali D., Thonart P. (2014). Lack of isocitrate lyase in Chlamydomonas leads to changes in carbon metabolism and in the response to oxidative stress under mixotrophic growth. Plant J..

[18] Johnson X., Alric J. (2012). Interaction between starch breakdown, acetate assimilation, and photosynthetic cyclic electron flow in *Chlamydomonas reinhardtii*. J. Biol. Chem..

[19] Wijffels R.H., Kruse O., Hellingwerf K.J. (2013). Potential of industrial biotechnology with cyanobacteria and eukaryotic microalgae. Curr. Opin. Biotechnol..

[20] Grossman A.R. (2000). *Chlamydomonas reinhardtii* and photosynthesis: Genetics to genomics. Curr. Opin. Plant Biol..

[21] Peltier G., Aro E.M., Shikanai T. (2016). NDH-1 and NDH-2 plastoquinone reductases in oxygenic photosynthesis. Annu. Rev. Plant Biol..

[22] Allen J.F. (2002). Photosynthesis—Telling it like it is. Trends Plant Sci..

[23] Dietz K.-J., Turkan I., Krieger-Liszkay A. (2016). Redox- and reactive oxygen species-dependent signaling into and out of the photosynthesizing chloroplast. Plant Physiol..

[24] Ledford H.K., Niyogi K.K. (2005). Singlet oxygen and photo-oxidative stress management in plants and algae. Plant Cell Environ..

[25] Erickson E., Wakao S., Niyogi K.K. (2015). Light stress and photoprotection in *Chlamydomonas reinhardtii*. Plant J..

[26] Curien G., Flori S., Villanova V., Magneschi L., Giustini C., Forti G., Matringe M., Petroutsos D., Kuntz M., Finazzi G. (2016). The water to water cycles in microalgae. Plant Cell Physiol..

[27] Saroussi S., Sanz-Luque E., Kim R.G., Grossman A.R. (2017). Nutrient scavenging and energy management: Acclimation responses in nitrogen and sulfur deprived Chlamydomonas. Curr. Opin. Plant Biol..

[28] Dang K.-V., Plet J., Tolleter D., Jokel M., Cuiné S., Carrier P., Auroy P., Richaud P., Johnson X., Alric J. (2014). Combined increases in mitochondrial cooperation and oxygen photoreduction compensate for deficiency in cyclic electron flow in *Chlamydomonas reinhardtii*. Plant Cell.

[29] Tolleter D., Ghysels B., Alric J., Petroutsos D., Tolstygina I., Krawietz D., Happe T., Auroy P., Adriano J.M., Beyly A. (2011). Control of hydrogen photoproduction by the proton gradient generated by cyclic electron flow in *Chlamydomonas reinhardtii*. Plant Cell.

[30] Desplats C., Mus F., Cuine S., Billon E., Cournac L., Peltier G. (2009). Characterization of Nda2, a plastoquinone-reducing Type II NAD(P)H dehydrogenase in Chlamydomonas chloroplasts. J. Biol. Chem..

[31] Peltier G., Cournac L. (2002). Chlororespiration. Annu. Rev. Plant Biol..

[32] Jans F., Mignolet E., Houyoux P.-A., Cardol P., Ghysels B., Cuiné S., Cournac L., Peltier G., Remacle C., Franck F. (2008). A type II NAD(P)H dehydrogenase mediates light-independent plastoquinone reduction in the chloroplast of Chlamydomonas. Proc. Natl. Acad. Sci. USA.

[33] Peltier G., Schmidt G.W. (1991). Chlororespiration: An adaptation to nitrogen deficiency in *Chlamydomonas Reinhardtii*. Proc. Natl. Acad. Sci. USA.

[34] Saroussi S.I., Wittkopp T.M. (2016). The Type II NADPH dehydrogenase facilitates cyclic electron flow, energy-dependent quenching, and chlororespiratory metabolism during acclimation of *Chlamydomonas reinhardtii* to nitrogen deprivation. Plant Physiol..

[35] Chen H., Hu J., Qiao Y., Chen W., Rong J., Zhang Y., He C., Wang Q. (2015). Ca^2+^-regulated cyclic electron flow supplies ATP for nitrogen starvation-induced lipid biosynthesis in green alga. Sci. Rep..

[36] Eisenhut M., Roell M.S. (2019). Mechanistic understanding of photorespiration paves the way to a new green revolution. New Phytol..

[37] Wang Y., Stessman D.J., Spalding M.H. (2015). The CO_2_ concentrating mechanism and photosynthetic carbon assimilation in limiting CO_2_: How Chlamydomonas works against the gradient. Plant J..

[38] Houille-Vernes L., Rappaport F., Wollman F.A., Alric J., Johnson X. (2011). Plastid terminal oxidase 2 (PTOX2) is the major oxidase involved in chlororespiration in Chlamydomonas. Proc. Natl. Acad. Sci. USA.

[39] Feilke K., Streb P., Cornic G., Perreau F., Kruk J., Krieger-Liszkay A. (2016). Effect of Chlamydomonas plastid terminal oxidase 1 expressed in tobacco on photosynthetic electron transfer. Plant J..

[40] Jokel M., Johnson X., Peltier G., Aro E.M., Allahverdiyeva Y. (2018). Hunting the main player enabling *Chlamydomonas reinhardtii* growth under fluctuating light. Plant J..

[41] Chaux F., Burlacot A., Mekhalfi M., Auroy P., Blangy S., Richaud P., Peltier G. (2017). Flavodiiron proteins promote fast and transient O_2_ photoreduction in Chlamydomonas. Plant Physiol..

[42] Saroussi S., Karns D.A.J., Thomas D.C., Bloszies C., Fiehn O. (2019). Alternative outlets for sustaining photosynthetic electron transport during dark-to-light transitions. Proc. Natl. Acad. Sci. USA.

[43] Badger M.R. (1985). Photosynthetic oxygen exchange. Annu. Rev. Plant Physiol..

[44] Jokel M., Kosourov S., Battchikova N., Tsygankov A.A., Aro E.M., Allahverdiyeva Y. (2015). Chlamydomonas flavodiiron proteins facilitate acclimation to anoxia during sulfur deprivation. Plant Cell Physiol..

[45] Franck F., Ghysels B., Godaux D. (2017). Hydrogen photoproduction by oxygenic photosynthetic microorganisms: Technologies and applications. Microbial Fuels.

[46] Melis A., Zhang L., Forestier M., Ghirardi M.L., Seibert M. (2000). Sustained photobiological hydrogen gas production upon reversible inactivation of oxygen evolution in the green alga *Chlamydomonas reinhardtii*. Plant Physiol..

[47] Hemschemeier A., Happe T. (2011). Alternative photosynthetic electron transport pathways during anaerobiosis in the green alga *Chlamydomonas reinhardtii*. Biochim. Biophys. Acta.

[48] Zhang L., Happe T., Melis A. (2002). Biochemical and morphological characterization of sulfur-deprived and H_2_-producing *Chlamydomonas reinhardtii* (green alga). Planta.

[49] Doebbe A., Keck M., La Russa M., Mussgnug J.H., Hankamer B., Tekce E., Niehaus K., Kruse O. (2010). The interplay of proton, electron, and metabolite supply for photosynthetic H_2_ production in *Chlamydomonas reinhardtii*. J. Biol. Chem..

[50] Hemschemeier A., Fouchard S., Cournac L., Peltier G., Happe T. (2008). Hydrogen production by *Chlamydomonas reinhardtii*: An elaborate interplay of electron sources and sinks. Planta.

[51] Chochois V., Dauvillee D., Beyly A., Tolleter D., Cuine S., Timpano H., Ball S., Cournac L., Peltier G. (2009). Hydrogen production in Chlamydomonas: Photosystem II-dependent and -independent pathways differ in their requirement for starch metabolism. Plant Physiol..

[52] Chochois V., Constans L., Dauvillee D., Beyly A., Soliveres M., Ball S., Peltier G., Cournac L. (2010). Relationships between PSII-independent hydrogen bioproduction and starch metabolism as evidenced from isolation of starch catabolism mutants in the green alga *Chlamydomonas reinhardtii*. Int. J. Hydrogen Energy.

[53] Mignolet E., Lecler R., Ghysels B., Remacle C., Franck F. (2012). Function of the chloroplastic NAD(P)H dehydrogenase NDA2 for H_2_ photoproduction in sulphur-deprived *Chlamydomonas reinhardtii*. J. Biotechnol..

[54] Fouchard S., Hemschemeier A., Caruana A., Pruvost J., Legrand J., Happe T., Peltier G., Cournac L. (2005). Autotrophic and mixotrophic hydrogen photoproduction in sulfur-deprived Chlamydomonas cells. Appl. Environ. Microbiol..

[55] Cardol P., Gloire G., Havaux M., Remacle C., Matagne R., Franck F. (2003). Photosynthesis and state transitions in mitochondrial mutants of *Chlamydomonas reinhardtii* affected in respiration. Plant Physiol..

[56] Uhmeyer A., Cecchin M. (2017). Impaired mitochondrial transcription termination disrupts the stromal redox poise in Chlamydomonas. Plant Physiol..

[57] Massoz S., Larosa V., Horrion B., Matagne R.F., Remacle C., Cardol P. (2015). Isolation of *Chlamydomonas reinhardtii* mutants with altered mitochondrial respiration by chlorophyll fluorescence measurement. J. Biotechnol..

[58] Kaye Y., Huang W., Clowez S., Saroussi S., Idoine A., Sanz-Luque E., Grossman A.R. (2019). The mitochondrial alternative oxidase from *Chlamydomonas reinhardtii* enables survival in high light. J. Biol. Chem..

[59] Matsuo M., Obokata J. (2006). Remote control of photosynthetic genes by the mitochondrial respiratory chain. Plant J..

[60] Noguchi K., Yoshida K. (2008). Interaction between photosynthesis and respiration in illuminated leaves. Mitochondrion.

[61] Peltier G., Thibault P. (1985). O_2_ uptake in the light in Chlamydomonas: Evidence for persistent mitochondrial respiration. Plant Physiol..

[62] Bailleul B., Berne N., Murik O., Petroutsos D., Prihoda J., Tanaka A., Villanova V., Bligny R., Flori S., Falconet D. (2015). Energetic coupling between plastids and mitochondria drives CO_2_ assimilation in diatoms. Nature.

[63] Merchant S.S., Prochnik S.E., Vallon O., Harris E.H., Karpowicz S.J., Witman G.B., Terry A., Salamov A., Fritz-Laylin L.K., Marechal-Drouard L. (2007). The Chlamydomonas genome reveals the evolution of key animal and plant functions. Science.

[64] Lemaire S.D., Quesada A., Merchan F., Corral J.M., Igeno M.I., Keryer E., Issakidis-Bourguet E., Hirasawa M., Knaff D.B., Miginiac-Maslow M. (2005). NADP-malate dehydrogenase from unicellular green alga *Chlamydomonas reinhardtii*. A first step toward redox regulation?. Plant Physiol..

[65] Tardif M., Atteia A., Specht M., Cogne G., Rolland N., Brugière S., Hippler M., Ferro M., Bruley C., Peltier G. (2012). PredAlgo, a new subcellular localization prediction tool dedicated to green algae. Mol. Biol. Evol..

[66] Hayashi Y., Shinozaki A. (2012). Visualization of microbodies in *Chlamydomonas Reinhardtii*. J. Plant Res..

[67] Kong F., Liang Y., Legeret B., Beyly-Adriano A., Blangy S., Haslam R.P., Napier J.A., Beisson F., Peltier G., Li-Beisson Y. (2017). Chlamydomonas carries out fatty acid beta-oxidation in ancestral peroxisomes using a bona fide acyl-CoA oxidase. Plant J..

[68] Lauersen K.J., Willamme R., Coosemans N., Joris M., Kruse O., Remacle C. (2016). Peroxisomal microbodies are at the crossroads of acetate assimilation in the green microalga *Chlamydomonas Reinhardtii*. Algal Res..

[69] Lemaire C., Wollman F.A., Bennoun P. (1988). Restoration of phototrophic growth in a mutant of *Chlamydomonas reinhardtii* in which the chloroplast atpB gene of the ATP synthase has a deletion: An example of mitochondria-dependent photosynthesis. Proc. Natl. Acad. Sci. USA.

[70] Liang Y., Kong F., Torres Romero I., Burlacot A., Cuine S., Legeret B., Billon E., Brotman Y., Alseekh S., Fernie A.R. (2019). Branched-chain amino acid catabolism impacts triacylglycerol homeostasis in *Chlamydomonas reinhardtii*. Plant Physiol..

[71] Salomé P.A., Merchant S.S. (2019). A series of fortunate events: Introducing Chlamydomonas as a reference organism. Plant Cell.

[72] Grossman A. (2000). Acclimation of *Chlamydomonas reinhardtii* to its nutrient environment. Protist.

[73] Kong F., Burlacot A., Liang Y., Legeret B., Alseekh S., Brotman Y., Fernie A.R., Krieger-Liszkay A., Beisson F., Peltier G. (2018). Interorganelle communication: Peroxisomal MALATE DEHYDROGENASE 2 connects lipid catabolism to photosynthesis through redox coupling in Chlamydomonas. Plant Cell.

[74] Kong F., Romero I.T., Warakanont J., Li-Beisson Y. (2018). Lipid catabolism in microalgae. New Phytol..

[75] Johnson X. (2011). Manipulating RuBisCO accumulation in the green alga, *Chlamydomonas reinhardtii*. Plant Mol. Biol..

[76] Siaut M., Cuiné S., Cagnon C., Fessler B., Nguyen M., Carrier P. (2011). Oil accumulation in the model green alga *Chlamydomonas reinhardtii*: Characterization, variability between common laboratory strains and relationship with starch reserves. BMC Biotechnol..

[77] Fan J.L., Andre C., Xu C.C. (2011). A chloroplast pathway for the de novo biosynthesis of triacylglycerol in *Chlamydomonas Reinhardtii*. FEBS Lett..

[78] Ball S., Dirick L., Decq A., Martiat J., Matagne R. (1990). Physiology of starch storage in the monocellular alga *Chlamydomonas reinhardtii*. Plant Sci..

[79] Tunçay H., Findinier J., Duchêne T., Cogez V., Cousin C., Peltier G., Ball S.G., Dauvillée D. (2013). A forward genetic approach in *Chlamydomonas reinhardtii* as a strategy for exploring starch catabolism. PLoS ONE.

[80] Klein U. (1987). Intracellular carbon partitioning in *Chlamydomonas reinhardtii*. Plant Physiol..

[81] Li Y., Han D., Hu G., Dauvillee D., Sommerfeld M., Ball S., Hu Q. (2010). Chlamydomonas starchless mutant defective in ADP-glucose pyrophosphorylase hyper-accumulates triacylglycerol. Metab. Eng..

[82] Juergens M.T., Disbrow B., Shachar-Hill Y. (2016). The relationship of triacylglycerol and starch accumulation to carbon and energy flows during nutrient deprivation in *Chlamydomonas Reinhardtii*. Plant Physiol..

[83] Ruuska S.A., Girke T., Benning C., Ohlrogge J.B. (2002). Contrapuntal networks of gene expression during Arabidopsis seed filling. Plant Cell.

[84] Perez-Perez M.E., Mauries A., Maes A., Tourasse N.J., Hamon M., Lemaire S.D., Marchand C.H. (2017). The deep thioredoxome in *Chlamydomonas reinhardtii*: New insights into redox regulation. Mol. Plant.

[85] Li-Beisson Y., Shorrosh B., Beisson F., Andersson M., Arondel V., Bates P., Baud S., Bird D., DeBono A., Durrett T., Last R. (2010). Acyl lipid metabolism. The Arabidopsis Book.

[86] Salie M.J., Thelen J.J. (2016). Regulation and structure of the heteromeric acetyl-CoA carboxylase. Biochim. Biophys. Acta.

[87] Wu J., Sun Y., Zhao Y., Zhang J., Luo L., Li M., Wang J., Yu H., Liu G., Yang L. (2015). Deficient plastidic fatty acid synthesis triggers cell death by modulating mitochondrial reactive oxygen species. Cell Res..

[88] Zhao Y., Luo L., Xu J., Xin P., Guo H., Wu J., Bai L., Wang G., Chu J., Zuo J. (2018). Malate transported from chloroplast to mitochondrion triggers production of ROS and PCD in Arabidopsis thaliana. Cell Res..

[89] Yao L., Shen H., Wang N., Tatlay J., Li L., Tan T.W., Lee Y.K. (2017). Elevated acetyl-CoA by amino acid recycling fuels microalgal neutral lipid accumulation in exponential growth phase for biofuel production. Plant Biotechnol. J..

[90] Shtaida N., Khozin-Goldberg I., Solovchenko A., Chekanov K., Didi-Cohen S., Leu S., Cohen Z., Boussiba S. (2014). Downregulation of a putative plastid PDC E1α subunit impairs photosynthetic activity and triacylglycerol accumulation in nitrogen-starved photoautotrophic *Chlamydomonas reinhardtii*. J. Exp. Bot..

[91] Avidan O., Pick U. (2015). Acetyl-CoA synthetase is activated as part of the PDH-bypass in the oleaginous green alga *Chlorella desiccata*. J. Exp. Bot..

[92] De Marcos Lousa C., van Roermund C.W.T., Postis V.L.G., Dietrich D., Kerr I.D., Wanders R.J.A., Baldwin S.A., Baker A., Theodoulou F.L. (2013). Intrinsic acyl-CoA thioesterase activity of a peroxisomal ATP binding cassette transporter is required for transport and metabolism of fatty acids. Proc. Natl. Acad. Sci. USA.

[93] Fulda M., Schnurr J., Abbadi A., Heinz E., Browse J. (2004). Peroxisomal acyl-CoA synthetase activity is essential for seedling development in *Arabidopsis thaliana*. Plant Cell.

[94] Jessen D., Roth C., Wiermer M., Fulda M. (2015). Two activities of long-chain acyl-coenzyme A synthetase are involved in lipid trafficking between the endoplasmic reticulum and the plastid in Arabidopsis. Plant Physiol..

[95] Kim S., Yamaoka Y., Ono H., Kim H., Shim D., Maeshima M., Martinoia E., Cahoon E.B., Nishida I., Lee Y. (2013). AtABCA9 transporter supplies fatty acids for lipid synthesis to the endoplasmic reticulum. Proc. Natl. Acad. Sci. USA.

[96] Footitt S., Slocombe S.P., Larner V., Kurup S., Wu Y.S., Larson T., Graham I., Baker A., Holdsworth M. (2002). Control of germination and lipid mobilization by COMATOSE, the Arabidopsis homologue of human ALDP. EMBO J..

[97] Zauner S., Jochum W., Bigorowski T., Benning C. (2012). A cytochrome b_5_-containing plastid-located fatty acid desaturase from *Chlamydomonas reinhardtii*. Eukaryot. Cell.

[98] Yang W., Wittkopp T.M., Li X., Warakanont J., Dubini A., Catalanotti C., Kim R.G., Nowack E.C.M., Mackinder L.C.M., Aksoy M. (2015). Critical role of *Chlamydomonas reinhardtii* ferredoxin-5 in maintaining membrane structure and dark metabolism. Proc. Natl. Acad. Sci. USA.

[99] Schulz-Raffelt M., Chochois V., Auroy P., Cuiné S., Billon E., Dauvillée D., Li-Beisson Y., Peltier G. (2016). Hyper-accumulation of starch and oil in a Chlamydomonas mutant affected in a plant-specific DYRK kinase. Biotechnol. Biofuels.

[100] Salinas T., Larosa V., Cardol P., Marechal-Drouard L., Remacle C. (2014). Respiratory-deficient mutants of the unicellular green alga Chlamydomonas: A review. Biochimie.

[101] Du Z.-Y., Lucker B.F., Zienkiewicz K., Miller T.E., Zienkiewicz A., Sears B.B., Kramer D.M., Benning C. (2018). Galactoglycerolipid lipase PGD1 is involved in thylakoid membrane remodeling in response to adverse environmental conditions in Chlamydomonas. Plant Cell.

[102] Lauersen K.J., Kruse O., Mussgnug J.H. (2015). Targeted expression of nuclear transgenes in *Chlamydomonas reinhardtii* with a versatile, modular vector toolkit. Appl. Microbiol. Biotechnol..

[103] Crozet P., Navarro F.J., Willmund F., Mehrshahi P., Bakowski K., Lauersen K.J., Perez-Perez M.E., Auroy P., Gorchs Rovira A., Sauret-Gueto S. (2018). Birth of a photosynthetic chassis: A MoClo toolkit enabling synthetic biology in the microalga *Chlamydomonas reinhardtii*. ACS Synth. Boil..

[104] Baier T., Wichmann J., Kruse O., Lauersen K.J. (2018). Intron-containing algal transgenes mediate efficient recombinant gene expression in the green microalga *Chlamydomonas reinhardtii*. Nucleic Acids Res..

